# Metallic Nanoparticles Applications in Neurological Disorders: A Review

**DOI:** 10.1155/ijbm/4557622

**Published:** 2025-07-06

**Authors:** Ernesto Ibarra-Ramírez, Melissa Montes, Roger Alexei Urrutia, Diego Reginensi, Edwin A. Segura González, Luis Estrada-Petrocelli, Alexandra Gutierrez-Vega, Abhishek Appaji, Jay Molino

**Affiliations:** ^1^Faculty of Biosciences and Public Health, Specialized University of the Americas (UDELAS), Panama City, Panama; ^2^Faculty of Engineering, Latin University of Panama (ULATINA), Panama City, Panama; ^3^Faculty of Engineering, Architecture and Design, Interamerican University of Panama (UIP), Panama City, Panama; ^4^Faculty of Electrical Engineering, Technological University of Panama (UTP), Panama City, Panama; ^5^School of Medicine, University of Panama (UP), Panama City, Panama; ^6^National Secretariat of Science, Technology and Innovation (SENACYT)—National Research System (SNI), Panama City, Panama; ^7^School of Industrial Technology, Specialized Higher Technical Institute (ITSE), Panama City, Panama; ^8^Department of Biomedical Engineering, University of Arkansas, Fayetteville, Arkansas, USA; ^9^Department of Medical Electronics Engineering, B.M.S. College of Engineering, Bengaluru, India

**Keywords:** biomedical engineering, gold nanoparticles, magnetic nanoparticles, metallic nanoparticles, neurological disorders, silver nanoparticles, tissue regeneration, titanium nanoparticles

## Abstract

Metallic nanoparticles (NPs) possess unique physicochemical properties that have enabled their engineering for loading drugs, contrast agents, and targeting moieties for cellular and intracellular components, highlighting their emerging role as versatile tools in managing neurological disorders. In therapeutic applications, the surface plasmon resonance characteristics of gold and silver NPs and the responsiveness of magnetic nanoparticles (MNPs) to external magnetic fields facilitate the disruption of protein aggregates and the eradication of cancer cells. For diagnostic purposes, the inherent high electron density of metallic NPs makes them effective contrast agents in imaging technologies. Moreover, these NPs have proven their capability to traverse the blood–brain barrier (BBB) and interact with central nervous system (CNS) components. Despite their extensive scientific exploration and promising applications, metallic NPs have not yet achieved widespread clinical implementation, especially in comparison to polymer-based NPs. This article presents an in-depth examination of the physicochemical properties of metallic NPs relevant to neurological applications. It summarizes their roles in diagnosis and therapy, focusing on gold, magnetic, silver, titanium, and cerium NPs. Additionally, this document explains the incorporation of metal NPs in their application and their effect on the human body.

## 1. Introduction

Metallic nanoparticles (NPs), typically with sizes between 10 and 100 nm, exhibit specialized optical, thermal, and magnetic qualities. These properties enable them to develop advanced systems that could potentially revolutionize how neurological disorders are managed in clinical practices, offering new solutions to existing treatment challenges [1, 2].

Metallic NPs exhibit unique physicochemical properties that differentiate them significantly from conventional drug delivery systems. These properties include their high surface-to-volume ratio, plasmonic behavior, and customizable surface functionalization, allowing precise control over drug release kinetics and site-specific targeting. For instance, magnetic nanoparticles (MNPs) such as gold nanoparticles (AuNPs) and silver nanoparticles (AgNPs) can be surface-engineered to enhance their interaction with specific cellular receptors, improving cellular uptake and therapeutic efficacy. These aspects are comprehensively discussed in the chapter *Metallic Nanoparticulate Delivery Systems* by Ref. [3]. Furthermore, MNPs can evade efflux pumps in resistant cells, increasing drug retention and bioavailability [4]. These NPs also enable synergistic effects when combined with therapeutic agents, reducing required dosages and minimizing off-target toxicity [5]. These distinctive features of MNPs highlight their transformative potential in creating highly efficient, multifunctional drug delivery systems.

A wide range of neurological disorders, which encompass both sporadic and hereditary conditions, includes brain cancer, neurodegenerative diseases such as Alzheimer's and Parkinson's, multiple sclerosis (MS), and stroke. These conditions can present with symptoms that range from mild to severe and may lead to rapid deterioration and death in some instances. The pathology of these disorders typically involves processes such as protein aggregation, leading to neurodegeneration, dysregulation of immune responses, or the progressive degradation of neuronal structure and function. These processes are often associated with developmental abnormalities in the brain that culminate in neuronal death [3–8]. Despite ongoing research, diagnosis, and treatment, strategies for neurological disorders remain primarily ineffective, mainly due to the complex nature of the nervous system, which requires high precision and extensive expertise for successful intervention [5–10].

The blood–brain barrier (BBB), a highly selective permeable barrier within humans, establishes an essential separation between the vascular system and the central nervous system (CNS). This barrier significantly impedes the successful management and identification of neurological conditions [5, 8, 9, 11, 12]. In vitro, numerous therapeutic agents have shown promise, yet they fail to halt disease progression in vivo due to the limiting characteristics of the BBB. Similarly, in diagnostic imaging, the specificity and sensitivity of contrast agents are often compromised as they rely on accumulation in areas where the BBB is weakened [9, 10, 12, 13].

Research highlights that metallic NPs with tailored surface modifications can traverse the BBB. Animal models have demonstrated that these NPs can infiltrate and accumulate within critical regions of the brain parenchyma, such as the striatum and hippocampus. This ability underscores the potential of metallic NPs as a promising tool for neurological interventions, offering new avenues for therapeutic and diagnostic applications. These NPs facilitate enhanced drug delivery directly to the brain and improve diagnostic imaging by providing better visualization of CNS structures, thereby addressing some of the significant challenges posed by the BBB in the management of neurological diseases [9, 11–15].

This review article aims to systematically evaluate recent research on NPs and their utilization in diagnosing and managing neurological disorders. This assessment focuses on the studies conducted over the past few years, highlighting these NPs' advancements and potential clinical uses. This study offers a comprehensive review of the BBB key characteristics; underscores the importance of research on brain tumors, Alzheimer's disease (AD), and Parkinson's disease (PD); and examines the properties of metallic NPs relevant to these neurological conditions. Additionally, we have focused on specific metallic NPs—gold, silver, iron-based, cerium, and titanium—summarizing significant research to develop new clinical diagnostics and treatments for neurological disorders.

## 2. Characteristics of the BBB

**Figure 1:**
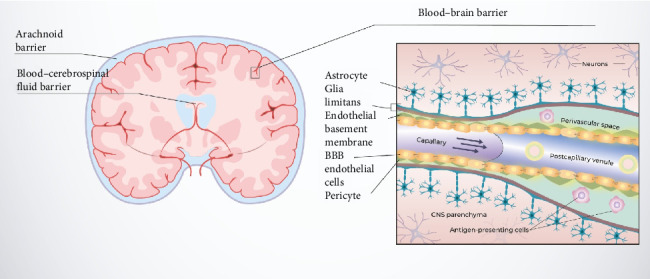
The coronal section of the brain shows the locations of the CNS's primary barriers, with particular emphasis on the BBB closest to the brain parenchyma (adapted from [14, 17]).

Three primary barriers isolate the CNS from the vascular system, forming a rigorously controlled structure. These barriers include the arachnoid membrane barrier, the barrier between blood and cerebrospinal fluid, and the BBB. The BBB is very close to the brain parenchyma, making it the most critical for accessing the CNS for neurological diagnostics and therapeutic drug delivery [8, 11, 13, 16]. A representation of the BBB in a coronal section is shown in Figure 1.

The BBB, a vast and intricate entity between the brain and blood, establishes and sustains a rigorously regulated microenvironment essential for proper neurotransmission; it functions as a signaling interface with a selective physical and metabolic transport system [15, 16, 18, 19]. The BBB consists of a single layer of endothelial cells forming the walls of brain capillaries, which are interconnected by tight junctions via the interaction of cell adhesion molecules like claudin and occludin and adherens junction molecules and gap junctions. Preserving the integrity of the BBB is vital for the CNS to function optimally. The BBB controls ionic and fluid dynamics to maintain conditions conducive to neuronal activity. It provides essential nutrients to the brain, distinguishes neurotransmitters in the CNS from those in peripheral areas, allows independent action, and supports immune surveillance and responses with minimal inflammation. These attributes define the BBB more as a dynamic interface than as a static barrier [8, 11, 15, 17, 18, 20]. Gap junctions among the endothelial cells support intercellular communication, while tight junctions and adherens junctions manage the permeability properties of the endothelium.

Receptor-mediated transcytosis (RMT) represents a pivotal mechanism for NPs to traverse the BBB efficiently. This process leverages specific ligand–receptor interactions to facilitate NP internalization into endothelial cells and subsequent release into the brain parenchyma. Commonly targeted receptors include the transferrin receptor (TfR), low-density lipoprotein receptor (LDLR), and insulin receptor (InsR), which are highly expressed on brain endothelial cells. As reviewed in Smart Strategies for Therapeutic Agent Delivery into Brain Across the Blood-Brain Barrier Using Receptor-Mediated Transcytosis by [21], this strategy has shown promise for transporting drugs to the brain. For instance, NPs conjugated with transferrin or ApoE have demonstrated enhanced transcytosis and subsequent accumulation in brain tissues [22]. Additionally, emerging strategies such as leveraging endothelial receptor recycling pathways and tuning NP surface properties to mimic endogenous molecules have further enhanced NP transcytosis efficiency [23]. These advancements underscore the importance of RMT as a transformative approach for delivering therapeutics to the CNS.

**Figure 2:**
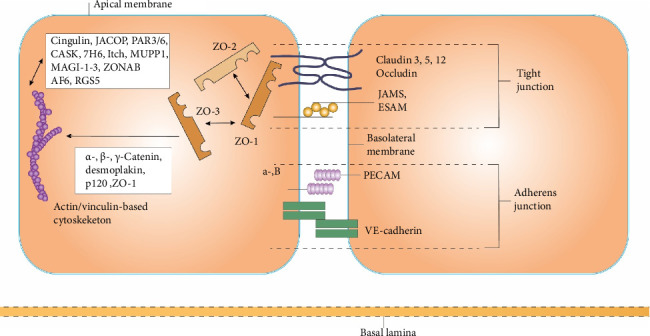
Claudin, occludin, JAM, and adherens junction proteins such as cadherin and PECAM. The diagram additionally depicts the apicobasal polarity and a glycocalyx bound to the luminal surface [8, 11].

Moreover, elements like the apicobasal polarity and the glycocalyx attached to the luminal surface are essential for the gateway function of the BBB [8, 11, 17, 20]. The localization and physiological roles of these endothelial junction proteins are further illustrated in Figure 2.

### 2.1. Tight Junction Complex

The composition of the tight junction complex primarily includes membrane proteins like claudins, occludins, and junctional adhesion molecules (JAMs), and it is further supported by additional cytoplasmic proteins such as ZO-1, ZO-2, ZO-3, and cingulin. These junctional proteins in endothelial cells' apical/luminal area effectively seal intercellular gaps. In contrast, auxiliary proteins link the junction proteins to intracellular cytoskeletal elements, critical for the selective permeability of the BBB. Despite their structural importance, tight junctions are dynamic and adapt to various stimuli, which highlights their potential role in diagnostics and therapeutic manipulation of the BBB [18, 24].

Various claudin proteins, specifically claudin 3, 5, and 12, form dimers that link to claudin molecules from adjacent endothelial cells, thereby establishing the primary intercellular seal within tight junctions. The carboxy-terminus of these claudins interacts with auxiliary cytoplasmic proteins such as ZO-1, ZO-2, and ZO-3 [18, 19, 24, 25]. Additionally, seven types of occludins have been identified, characterized by their 11 tyrosine and 19 glycine residues, which confer structural flexibility. Unlike claudins, occludins have their intracellular segments closely associated with ZO proteins. They support tight junction integrity and act as signal transducers in response to cytokines during inflammatory responses [8, 11, 20, 26]. JAMs, including JAM-1, JAM-2, and JAM-3, do not participate directly in forming tight junctions but are integral in assembling tight junction components and adjusting the complex that maintains endothelial polarization [17, 20, 21, 27].

### 2.2. Adherens Junction Complex

The adherens junction complex is made up of multiple essential elements, including cadherins, platelet–endothelial cell adhesion molecule (PECAM), and different types of catenins (α-, β-, and γ-catenin), as well as desmoplakin and p120 catenin. Cadherins facilitate connections with neighboring cells using a calcium-dependent mechanism and serve three primary functions: establishing links with adjacent endothelial cells, supporting vascular growth, and promoting the polarization process of endothelial cells [22, 23, 28, 29]. Cadherin-5, also known as vascular endothelial cadherin (VE-cadherin), is essential for maintaining microvascular integrity, with overexpression linked to reduced cell proliferation and decreased cell permeability and migration. Additionally, PECAM, catenins, desmoplakin, and p120 catenin are vital for anchoring transmembrane glycoproteins to the cytoskeleton, critical to cellular adhesion and integrity [23, 29].

### 2.3. BBB Polarization and Neurological Function

In the CNS, apicobasal polarity is more distinct compared to other regions of the body due to several factors: (i) the apical/luminal and basolateral/abluminal plasma membranes exhibit considerable differences in their lipid and glycoprotein composition, fluidity, surface charge, and lipid arrangement; (ii) the distribution of receptors varies across the apical and basolateral membranes; (iii) substances such as platelet-derived growth factor (PDGF) are predominantly secreted from the basolateral side in a polarized fashion; (iv) there is a polarized reaction to environmental stimuli [8, 11]. Moreover, the luminal surface of microvessel endothelia is enveloped by a glycocalyx. This carbohydrate-rich layer binds to endothelial cells via glycoproteins and proteoglycans, playing a critical role in safeguarding and supporting the BBB by sequestering potentially neurotoxic molecules and aiding in the cellular uptake of various substances [13, 16, 24, 30].

**Figure 3:**
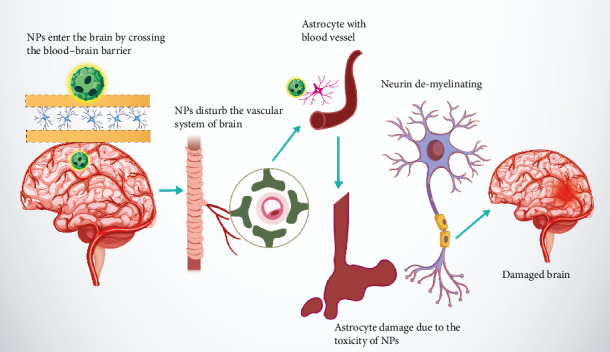
Mechanism of nanoparticle (NP) neurotoxicity: Nanoparticles cross the blood–brain barrier, disturb vascular systems, damage astrocytes, and cause neuronal demyelination, resulting in brain damage.

When neurotoxic, NPs cross the BBB, disrupting the brain's vascular system and damaging astrocytes in proximity to blood vessels. This toxicity leads to neuronal demyelination, impairing neural function and ultimately causing visible damage to brain structure.  Figure 3 emphasizes the potential risks associated with NP exposure and their impact on neural health.

The polarity of the BBB governs the expression and function of proteins found in intercellular junctions. Conversely, endothelial junctions are crucial for maintaining this polarity, demonstrating a bidirectional relationship that ensures the BBB's optimal functionality. These junctions provide anchoring points that support cellular polarity, which controls the junction proteins' expression and operation [17, 20, 25, 31].

## 3. Neurological Disorders

Neurological disorders encompass a broad spectrum, linked to over 600 distinct diseases, including epilepsy, dementia, AD, and cerebrovascular disorders such as stroke, MS, PD, migraines, neuroinfections, brain tumors, and trauma-related conditions like brain injury and autism [7, 10]. These conditions are notably diverse in their origins, ranging from pathological protein aggregation leading to neurodegeneration, dysregulation of immune responses, and developmental and functional anomalies in the brain [26, 32]. The complexity of these disorders often stems from intricate interactions between genetic predispositions and environmental factors [6, 9].

Accurate and swift diagnosis is crucial for effectively managing these conditions and significantly enhancing patient outcomes. Various sophisticated diagnostic tools are employed extensively to assist in detecting, managing, and treating neurological disorders. Imaging methods such as magnetic resonance imaging (MRI), computed tomography (CT), positron emission tomography (PET), and angiography are used alongside signal-based techniques like electroencephalography (EEG) and electromyography (EMG). Ongoing research continues to refine these technologies, ensuring they remain at the forefront of medical practice for neurological conditions [7, 10, 27, 33].

### 3.1. Brain Tumor

Brain tumors are characterized by the abnormal accumulation of cells within the brain that grow uncontrollably [7, 10, 28, 34]. These tumors vary widely, encompassing many different types, each with unique characteristics. According to the World Health Organization (WHO), the classification system for CNS tumors includes more than 100 histologic types based on cell origin and other histopathologic features [29, 30, 35, 36]. Brain tumors can be benign (noncancerous) or malignant (cancerous). They may develop directly in the brain (referred to as primary brain tumors) or migrate from other regions of the body to the brain (known as secondary or metastatic brain tumors). Reports indicate that 1 in 50 individuals under the age of 60 will die from a brain tumor [7, 10]. The most common benign tumors are meningiomas, while gliomas are the most prevalent malignant type, with glioblastoma appearing most frequently [29, 35]. Recent years have seen a rise in the occurrence of this type of cancer. Globally, the incidence and mortality rates are documented at 3.4 and 2.5 per 100,000 individuals, respectively, although these numbers differ from one region to another. Significant risk factors for brain cancer include radiation exposure, hormonal influences, family history, immune deficiencies, nutritional factors, alcohol consumption, cigarette smoking, aspartame intake, and cell phone usage [31, 37].

Diagnosing brain tumors involves appropriate imaging and histopathological examination. Gadolinium-enhanced MRI is often the modality of choice due to its high resolution and effective use of contrast agents. When MRI is not feasible, such as in patients with metallic implants, embedded devices, or claustrophobia, head and spine CT scans serve as alternatives, although they offer lower resolution and are less effective in assessing posterior fossa and spine lesions. Since these techniques cannot detect lesions smaller than 3 mm, specialized brain tumor segmentation methods are typically utilized in medical diagnostics.

Advanced imaging techniques for brain tumor segmentation have significantly improved precision in identifying tumor boundaries, volume, and shape. Volumetric analysis methods, such as 3D convolutional neural networks like U-Net and ResUNet, enable segmentation directly from MRI volumetric data, achieving high accuracy for delineating tumor regions by leveraging deep learning architectures [38]. Hybrid algorithms, such as active contour models combined with region-growing techniques, address challenges posed by irregular tumor shapes and inhomogeneities in MRI data, offering robust tumor boundary detection [39]. Additionally, 3D cross-modality approaches, such as ACMINet, integrate multimodal MRI data, enhancing the accuracy of tumor tissue segmentation by dynamically aligning low-level and high-level image features [40]. These techniques collectively enable more precise visualization and quantification of tumor characteristics, aiding clinicians in treatment planning and prognosis.

Techniques such as perfusion MRI, magnetic resonance spectroscopy, and fluorodeoxyglucose PET may also be required for comprehensive diagnosis and staging [28, 32–34, 41, 42]. Locating the primary site of a tumor is often challenging without distinct clinical indicators from the patient's history and physical examination. The precision of imaging may be further limited by unusual cases or the limited clinical experience of the radiologist [7, 10, 32, 41]. Additionally, while the classification of brain tumors is traditionally based on microscopic morphology and immunohistochemistry, this approach does not fully incorporate genomic subtyping, which is crucial for precision medicine. Ongoing debate exists on integrating and applying the extensive data collected to tailor treatment more effectively [34, 43].

Treatment decisions for brain cancer are influenced by the type and location of the tumor, its potential for malignancy, and the patient's age and overall health. Options can range from active surveillance for less aggressive tumors to interventions such as surgery, radiotherapy, chemotherapy, or a combination thereof. Patients might also be offered participation in clinical trials for certain high-grade tumors [32, 41]. Aldape et al. have identified seven key challenges that need to be addressed to advance brain tumor therapy significantly. These challenges primarily revolve around a limited understanding of brain tumor biology and the tumor microenvironment underutilization of available research data, among other issues [34, 43]. There is an urgent need for more research aimed at developing new treatments that not only slow disease progression but also enhance symptom management [6, 9]. Despite extensive research efforts focused on diagnosing and treating brain tumors, only a few advances are being effectively translated into clinical practice.

Metallic nanoparticles (MNPs) offer unique capabilities for targeting disease-specific pathologies in AD and PD beyond conventional drug delivery systems. In AD, MNPs such as polydopamine-coated AuNPs (Au@PDA-Apt NPs) have been shown to inhibit the fibrillization of beta-amyloid (Aβ) peptides and disaggregate existing Aβ fibrils, effectively reducing amyloid toxicity and preventing cell membrane damage [44]. Additionally, multifunctional NPs capable of chelating metal ions such as copper, which exacerbate Aβ aggregation and oxidative stress, have demonstrated potential in restoring metal homeostasis and reducing Aβ-induced neurotoxicity [45]. In PD, specific copper chelators, such as TDMQ20, have shown promise in reducing alpha-synuclein (α-syn) aggregation, mitigating oxidative stress, and improving cognitive deficits in preclinical models of PD [46]. These advancements highlight the potential of MNPs as precise tools for modulating the pathological hallmarks of neurodegenerative diseases.

### 3.2. AD

**Figure 4:**
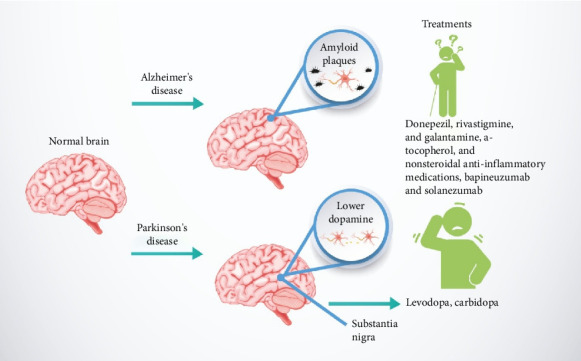
Schematic overview of the causes and general treatments for Alzheimer's and Parkinson's diseases.

AD is recognized as the most prevalent neurodegenerative disorder affecting the elderly and is notably linked to severe cognitive impairments. People with AD experience minor forgetfulness and difficulties with memory imprinting, which escalates to short-term memory loss and, eventually, severe long-term memory deficits. These cognitive issues progressively impair thinking, remembering, and reasoning abilities, leading to behavioral issues that disrupt daily life and activities. In advanced stages, individuals become completely dependent on others for basic daily tasks [11, 14, 27, 33]. AD affects approximately 1% of people aged 50 to 70, with the prevalence increasing to 50% after age 70 [35, 47]. The primary pathological characteristics of AD are characterized by the accumulation of amyloid deposits from clumped Aβ proteins and the development of neurofibrillary tangles within cells, which occur due to the clustering of excessively phosphorylated tau protein [36, 37, 48, 49]. Additionally, the progression of AD is marked by the loss of cholinergic neurotransmitters, specifically noted by decreased choline acetyltransferase activity leading to lower acetylcholine levels, which play a crucial role in memory and cognitive functions in the brain [38, 50].  Figure 4 illustrates AD in terms of its causes and treatments compared to PD, which will be discussed in the next subsection.

**Figure 5:**
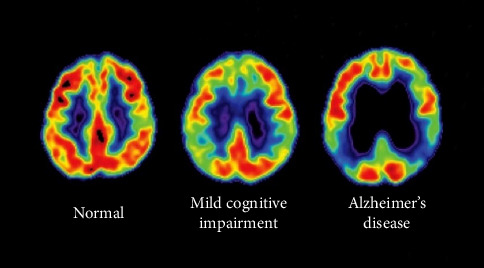
PET image of brain activity in AD, as compared with normal brain and mild cognitive impairment [39, 51].

The diagnosis of AD is mainly performed by clinical analysis of symptoms [36, 48] and automatic computer assistance methods for detecting AD using structural MRI data and PET scans [7, 10].  Figure 5 shows a PET image comparison of regular brain activity, mild cognitive impairment, and AD.

At present, AD remains incurable, with treatment strategies primarily focusing on symptom management and mitigation [35, 47]. The therapeutic regimen often includes pharmacological agents such as donepezil, rivastigmine, and galantamine, commonly used to enhance acetylcholine bioavailability by inhibiting its breakdown [40, 52]. Additionally, there are suggestions for using anti-inflammatory drugs and antioxidants, like α-tocopherol and nonsteroidal anti-inflammatory drugs, as part of the treatment approach. Recent research has also explored the potential of immunotherapy for breaking down Aβ plaques, with promising results achieved through monoclonal antibodies engineered to be compatible with human immune systems, such as bapineuzumab and solanezumab [41, 42, 53, 54].

### 3.3. PD

PD, classified as the second most prevalent progressive neurodegenerative disorder worldwide, has elusive origins and mechanisms. The characteristic motor symptoms of PD primarily originate from the loss of dopamine-producing cells in the substantia nigra, a critical area of the midbrain. This loss results in significant dopamine shortages, presenting clinically as bradykinesia, rigidity, and tremor [43, 44, 55, 56]. Moreover, genetic studies have implicated mutations in the α-synuclein protein in the development of Lewy bodies, which accumulate in several neuronal populations and disrupt various neurotransmitter systems, including adrenergic, cholinergic, and serotonergic pathways [11, 14, 45, 57].

Epidemiologically, PD primarily affects older adults, with prevalence rates, indicating that approximately one in every 200 people aged 60 to 69, one in 100 people aged 70 to 79, and one in 35 people aged 80 to 89 in more developed regions are living with PD [44, 56]. PD patients typically experience progressive impairments in motor skills, such as walking and balance, alongside symptoms like tremors, stiffness, and difficulty with coordination. These symptoms often start subtly and worsen over time [27, 33].

For both PD and AD, diagnosis primarily involves clinical symptom evaluation [46, 58] and is aided by advanced imaging technologies [7, 10]. Studies indicate that early detection of PD, before the onset of motor symptoms, might be possible using specialized ligands that target specific molecular markers for imaging. This approach quantifies gene and protein functions and provides deeper insights into PD's molecular pathophysiology, such as interactions and signaling pathways [44, 47, 48, 56, 59, 60].

Currently, no PD cure exists, and treatments are focused on managing symptoms. Therapies often include dopaminergic agonists such as levodopa, which acts like dopamine. Paired with carbidopa, which inhibits its peripheral metabolism, it effectively enhances dopamine levels in the brain. Nevertheless, the high oral decarboxylation rate of levodopa necessitates higher doses to be effective, which may lead to side effects, including depression, anxiety, insomnia, nausea, and vomiting [49, 61]. Surgical interventions, such as stimulation of the brain's inner regions and transplantation of fetal neurons, have been explored; the U.S. Food and Drug Administration (FDA) gave its approval for deep brain stimulation as a treatment method for PD in 2002; however, its application is currently rare [46, 50, 58, 62]. Despite ongoing research, many promising therapies have not yet become available for clinical application [27, 33].

## 4. Neurological Applications of Metallic NPs

Metallic NPs have gained significant attention in recent years for their distinct properties, particularly in clinical applications, due to their uniform size and precise size distribution [1]. Commonly utilized in medical applications, these NPs typically range from 10 to 500 nm in size, occasionally reaching up to 700 nm. Their nanoscale size facilitates effective interaction with external and internal biomolecules within cells, enabling them to influence cellular biochemical and physiochemical processes. This capability is precious in drug delivery systems and noninvasive imaging, providing advantages over traditional pharmaceutical agents [51, 63]. Metallic NPs are engineered to be stable, biocompatible, and capable of targeted delivery to specific body sites following systemic administration to enhance their clinical utility. Advanced targeting systems have also been developed to identify particular cells, including cancer cells, or, in the context of neurology, to traverse the BBB effectively [9, 12, 52, 64]. These advancements have vastly expanded the possibilities for diagnosing and treating neurological disorders [53, 65].

**Figure 6:**
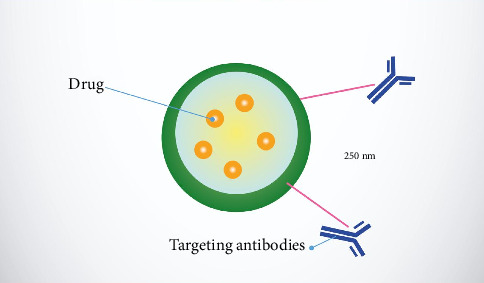
Polymeric nanoparticle surface modified for targeting antibodies.

Metallic NPs can be efficiently combined with different substances like peptides, antibodies, and DNA/RNA to aim at particular cells, along with biocompatible polymers such as polyethylene glycol (PEG) to prolong their presence in the bloodstream for improved medication and gene transport potential [54, 56, 66–68]. Upon reaching their target, these NPs can function as imaging agents in various methods, encompassing ultrasound (US), CT, PET, MRI, optical imaging, and surface-enhanced Raman scattering (SERS). This capability allows for the detailed visualization of cellular functions and monitoring of neurological processes in living organisms without disturbance [57, 58, 69, 70]. A diagram showing a schematic of a polymeric NP surface modified for targeting antibodies is presented in Figure 6.

In-depth studies and modifications continue to enhance the diagnostic and therapeutic capabilities of metallic NPs like magnetic (iron-based), AuNPs, AgNPs, and titanium NPs. The subsequent sections will discuss the specific properties of these NPs and their applications in neurological disorders.

### 4.1. MNPs

Given their ultrafine size, strong magnetization, and biocompatibility, MNPs are gaining recognition for their broad usefulness in various biomedical fields. Their effectiveness depends on maintaining strong magnetization, enabling their use in diverse roles like high-definition MRI contrast mediums, targeted medication delivery and imaging, hyperthermia procedures, gene treatments, stem cell monitoring, and molecular/cellular tracking. MNPs also play a pivotal role in magnetic separation technologies like rapid DNA sequencing and are instrumental in detecting diseases such as inflammation, cancer, diabetes, and atherosclerosis [51, 63]. In neurology, MNPs have shown the ability to disrupt the BBB temporarily, enhancing their potential for targeted drug delivery into the CNS [59, 71].

The properties of MNPs, such as size, shape, and coating, significantly influence their magnetic forces and tissue resistance, with these characteristics tailored based on the physiological attributes of the target area. Particles that are too small may not generate sufficient magnetic force, while larger particles might face excessive resistance in tissues [60–62, 72–74]. Typically, optimal magnetic field strengths for these applications range from 0.2 to 0.7 T, with gradients between 8 and 100 T/m, adjusted according to blood flow dynamics.

Despite their potential in CNS therapies, MNPs face significant challenges in vivo, particularly regarding the limitations of magnetic field strength and working distances. The strength of the external magnetic field decreases rapidly with distance, which limits the precision and efficiency of MNP targeting in deep-seated CNS disorders. For example, recent studies have highlighted the need for optimized magnetic gradients to achieve enhanced targeting and localization of MNPs in therapeutic applications [75]. Moreover, surface-engineered MNPs have shown promise in addressing variability in targeting efficacy by improving stability and reducing off-target effects, though challenges in reproducibility remain [76]. Additionally, uneven distribution and aggregation caused by inconsistent field applications further complicate their use in vivo, underscoring the importance of advanced engineering for magnetic field optimization [77]. These limitations emphasize the need for continued innovation of nanoplatforms and magnetic field applications to overcome these barriers.

However, in clinical settings, especially within the human brain, the application of MNPs is constrained by factors like working distance (30–50 cm from the magnet surface) and magnetic field strength limitations (8 T for adults, 4 T for children) due to safety considerations [60, 63, 72, 78]. Magnetic targeting tends to be more effective for surface-level targets or areas with slower blood flow [64, 79]. MNPs are typically manufactured as colloidal ferrofluids, where their stability is dependent on the balance of forces such as interactions of Van der Waals, dipole–dipole, steric, thermal, and electrostatic that take place between the particles and the molecules of the surrounding solvent. MNPs are often encapsulated with biocompatible polymers like PEG, polyethyleneimine, polylysine, or polyacrylic acid to enhance stability and prevent aggregation due to their large hydrophobic surface-to-volume ratio. This polymer layer is essential to minimize aggregation risks that could lead to safety concerns such as blood vessel clotting. To further mitigate these risks, superparamagnetic iron oxide MNPs (SPIONPs) are frequently utilized [59, 65, 71, 80].

SPIONPs, composed of iron oxide with a core of either Fe_3_O_4_ or γFe_2_O_3_, range in diameter from 50 to 100 nm; those smaller than 50 nm are classified as ultrasmall superparamagnetic iron oxide nanoparticles (USPIONPs). These NPs have been shown to effectively cross the BBB in both in vitro and in vivo models. Their transport across the BBB depends on their surface properties, specifically the coatings that stabilize them, and their penetration is enhanced by applied magnetic forces [59, 66, 71, 81]. Notably, Fe_3_O_4_, often used with alginate, is approved for human use by the U.S. FDA, and its metabolites are considered safe at administered doses with no reported side effects [67, 82].

MNPs have shown promise in addressing various CNS disorders. In brain cancer treatment, they enhance the effectiveness of chemotherapy and help deliver gene therapies or act as “nanosurgeons” to target and destroy malignant cells [59, 71] mechanically. For AD and PD, MNPs are utilized alongside advanced imaging methods like PET, MRI, X-ray, and SPECT to aid in the early detection and comprehensive analysis of these diseases [53, 65].

Specifically in brain tumor therapy, Etminan et al. utilized alginate-coated iron oxide core nanoparticles (alg-Fe_3_O_4_ NPs) tagged with BBB-penetrating G23 peptides as a nanocarrier for the anticancer drug doxorubicin (Dox). In their research, this nanocarrier successfully crossed the BBB and targeted glioblastoma in both lab and animal models, using a U87-luc2-bearing mice cell line. This system effectively reduced or eliminated tumors within 7 days and served as an MRI contrast agent for both in vitro and in vivo diagnostic applications [53, 65, 67, 82].

Su et al. successfully synthesized SPIONPs as a contrast agent in the study of AD diagnostics using MRI. These SPIONPs were functionalized with a DDNP carboxyl derivative that targets Aβ aggregates, ranging in size from 60 to 150 nm. The results indicated that DDNP-SPIONPs might be used to visualize Aβ plaques in transgenic mice, potentially aiding early AD diagnosis and monitoring therapeutic responses. The research team is still investigating this conjugated approach to confirm its efficacy in vivo [82–84].

Similarly, Su et al. studied using USPIONPs coupled with the Aβ peptide for in vivo MRI to detect amyloid deposits. This approach involved administering the NPs intravenously in transgenic mice and mannitol, showcasing the feasibility of a less invasive technique for studying AD pathogenesis. Given the previous safe use of USPIONPs in humans, this method shows promise for further development toward clinical application [82–85].

Despite numerous studies highlighting the potential of MNPs in diagnosing and treating neurological disorders, significant challenges remain in transitioning these innovations from the laboratory to clinical trials [60, 72]. Research has successfully demonstrated MNP functionalization, targeting, BBB penetration, and drug delivery; however, these advancements have not yet been applied in clinical settings [9, 12]. Key obstacles include the rapid decrease in magnetic field strength at greater depths within the body, which is essential when scaling up from small animal models to human trials [60, 63, 72, 78]. Additionally, the creation of a magnetic trap using only static magnetic fields has proven impossible [60, 66, 72, 81], and managing a large volume of magnetic particles to achieve therapeutic effects remains complex [60, 72]. Another significant issue is the retention of MNP properties once the external magnetic field is removed [63, 78]. Moreover, effective and safe methods to manage BBB selectivity for MNPs and ensure their recovery after application are still under investigation, posing critical challenges for clinical application [59, 71].

### 4.2. AuNPs

AuNPs exhibit several advantageous properties, such as a tunable size, expansive surface area-to-volume ratio, and the capacity for diverse surface modifications, coupled with a high level of biocompatibility. These features establish AuNPs as effective carriers for crossing the BBB and targeting brain tumors [10, 13]. AuNPs uniquely interact with light, attributed to localized surface plasmon resonance (LSPR).

LSPR, unique to metallic NPs like gold, enhances their utility in imaging and photothermal therapy. By absorbing and scattering light at specific wavelengths, LSPR generates localized heat, enabling precise targeted tissue ablation. Gold nanorods and nanostars, with tunable LSPR peaks in the near-infrared (NIR) window, demonstrate superior penetration depth and reduced damage to surrounding tissues, making them ideal for tumor-specific applications [86]. For example, photothermal therapy using gold nanostars has shown enhanced efficacy in tumor ablation through hyperthermia induced by NIR laser irradiation [87]. Furthermore, the functionalization of AuNPs with targeting ligands enables simultaneous imaging and therapeutic delivery, as demonstrated in studies highlighting their integration into hybrid nanoplatforms for multimodal cancer therapies [88, 89]. These advancements underscore the potential of LSPR-based therapies in enhancing precision medicine.

This resonance occurs when the metal's free electrons oscillate against the lattice under an electromagnetic field at specific light frequencies, leading to energy absorption and conversion into scattered light or heat [51, 63].

Additionally, the significant surface area of AuNPs facilitates their use in cutting-edge applications such as photoimaging and photothermal therapy [68, 90]. For photothermal treatment, the efficiency of AuNPs in converting light to heat is vital, especially with smaller NPs that absorb light and convert it to heat to destroy cells [69, 91, 92]. Conversely, larger NPs are preferred in photoimaging due to their greater surface area, enhancing imaging capabilities. This method is particularly innovative in cancer treatment, where targeted injection of millions of functionalized AuNPs into tumors enables them to bind specifically to cancer cells. This binding enhances visibility, assisting surgeons in differentiating between cancerous and healthy cells during surgical procedures [70, 93]. AuNPs must be explicitly designed to overcome the BBB for practical use in diagnostics and therapy. Research has established that AuNPs within the 20–120 nm size range can cross the BBB and accumulate in the brain, with those around 50 nm in diameter being particularly effective. Notably, 10-nm AuNPs have shown the ability to penetrate the BBB when administered intravenously, though the passage rate diminishes as the NP size increases.

Further studies have demonstrated that AuNPs as large as 120 nm can cross the BBB by applying techniques like MRI-guided focused ultrasound (MRgFUS) to permeabilize brain tumor regions. Additionally, NP size influences their toxicity, with extremely small NPs (about 1.5 nm) showing high cytotoxicity. In comparison, those larger than 15 nm are generally nontoxic, indicating an inverse relationship between size and BBB penetration efficiency and toxicity levels [71, 94].

Regarding their shape, NPs with a shell structure are generally more efficiently absorbed and less toxic compared to those with a rod-like shape. To improve bioavailability for brain imaging, AuNPs are frequently coated with biocompatible polymers such as dextran, PEG, or poly(vinylpyrrolidone) (PVP). These coatings help to prevent coagulation, ensure particle monodispersion, and extend the NPs' circulation within the system [72–74, 95–97].

AuNPs are extensively researched nanomaterials with many applications in diagnostics and therapeutics. Diagnostics are used to detect tumor markers, identify microbial pathogens, and enhance various imaging techniques. Therapeutically, AuNPs are applied in gene therapy, cancer treatment, and drug delivery systems [85, 98]. Their significant role in neurological disorders is particularly noted due to their low toxicity, enhanced CT imaging contrast, and ability to be functionalized with various chemotherapies or targeted ligands. Additionally, AuNPs improve the effectiveness of radiation therapy (RT) in vitro and in vivo [86, 99].

AuNPs exhibit several properties conducive to crossing the BBB, including small size, inertness to tissue, and nonionic nature [87, 88, 100, 101]. AuNPs coated with PEG are noted for their stability under physiological conditions and biocompatibility, possessing anti-biofouling properties that enable prolonged systemic circulation. This enhances their permeability and retention, facilitating greater uptake in brain tumors [86, 89–91, 99, 102–104].

Chiang et al. developed AuNPs targeted with transferrin peptide (Tfpep) and coated with PEG and a fluorescent tag prodrug, Pc4. These targeted NPs were compared with nontargeted AuNPs for delivering a photosensitizer to brain cancer cell lines [92, 105]. Both in vitro studies on human glioblastoma cells (U87) and in vivo studies using orthotopic xenografts in mice demonstrated that Tf conjugation significantly increased the uptake of AuNPs by glioblastoma cells compared to the nonconjugated AuNPs. Furthermore, these NPs specifically targeted brain tumor tissues, with minimal accumulation in other organs, indicating their potential for precise brain tumor therapy delivery and as a platform for noninvasive imaging [71, 92, 94, 105]. Additional enhancements included the attachment of an epidermal growth factor (EGF) peptide, which improved BBB penetration, resulting in higher and faster accumulation of these agents, specifically in brain tumor regions [71, 93, 94, 106].

AuNPs have also been explored for diagnosing PD, particularly for detecting the aggregation of α-synuclein, a protein potentially implicated in PD. Qin et al. examined the interaction of α-synuclein with positively charged poly(allylamine hydrochloride)-coated AuNPs. This interaction altered the α-synuclein structure, making it more susceptible to enzymatic attack and consistently altering its conformation, independent of the concentration of AuNPs [44, 56, 94, 107].

As detailed earlier and outlined in Table 1, AuNPs are recognized for their potential in diagnosing and treating neurological disorders. Nonetheless, there is a significant concern regarding their unintended health effects [68, 90]. Research into the side effects of AuNPs has presented mixed findings, particularly regarding their cytotoxicity and how toxicity varies with particle size [95–98, 132–135]. Issues such as these NPs' effectiveness and biodistribution are also under scrutiny [99–101, 136–138]. Thus, the debate continues regarding the clinical viability of AuNPs [68, 90], and discussions are ongoing about whether the benefits in therapeutic outcomes justify the costs of their synthesis [102, 139].

### 4.3. AgNPs

AgNPs have the advantage of having a higher relative abundance and lower cost than AuNPs. Still, their susceptibility to oxidation has restricted their use in developing biomaterials [75, 140]. AgNPs are suitable for various medical applications due to their special properties, which include high electrical conductivity, low sintering temperatures, and broad-spectrum antibacterial activity. This antimicrobial efficacy is primarily due to silver ions (Ag^+^), which are reactive with numerous surrounding molecules, contributing to their versatility in healthcare settings [76, 77, 141, 142].

Numerous studies in the literature highlight the neurotoxic effects of AgNPs. These effects include alterations in brain electrical activity [79, 143], enhanced BBB permeability [80, 144], and increased production of reactive oxygen species (ROS) [81, 145], among others. It has been shown through analytical microscopy studies that AgNPs are assimilated by microglia, forming on their surface a layer of nonreactive silver sulfide (Ag_2_S) that reduces neurotoxicity and brain inflammation [78, 124].

The Global Burden of Diseases (GBD), injuries, and risk factor study has updated the classification of major neurological disorders, including AD, PD, epilepsy, and cancers of the brain and nervous system. It reports that AD and other dementias are particularly prevalent, with case numbers ranging from 40.2 to 52.7 million, amounting to approximately 46.0 million [146]. Furthermore, recent studies have increasingly focused on AgNPs for their low toxicity, high stability, and especially their proven biocompatibility, making them promising for biomedical applications [147]. Yet, the potential toxicity of AgNPs on the CNS remains unclear.

Recent studies have highlighted the dose-dependent toxicity of AgNPs in vivo, particularly their effects on key organs such as the liver, kidneys, and spleen. For instance, [148] demonstrated that high doses of AgNPs induced significant histopathological changes, including congestion, hemorrhage, and apoptosis in hepatic and renal tissues, as well as oxidative stress and depletion of antioxidants such as glutathione. These findings emphasize the importance of comprehensive toxicity evaluations, particularly as AgNPs move toward clinical applications.

As engineered nanomaterials (ENMs), AgNPs have shown anti-inflammatory effects in the brain when administered via inhalation or oral routes in rat models. However, their toxicity varies depending on factors such as temperature, pH, quantity, and particle size [149–152]. Additionally, AgNPs can penetrate the BBB and navigate through neuronal connections in axons or dendrites [153, 154]. Table 1 illustrates how, for AD and PD, MNPs are integrated with various brain imaging techniques (X-ray, MRI, PET, SPECT, etc.) to enable early diagnosis and detailed pathology examination.

### 4.4. Titanium NPs

Titanium, naturally prevalent in the earth's crust and lithosphere, typically appears in four crystal phases, with rutile and anatase phases being the focus of extensive research due to their superior photoactivity. These phases exhibit advantageous properties, including optimal optical, electrical, and mechanical characteristics and effective photocatalytic and antibacterial capabilities. Furthermore, they are recognized for their low toxicity, chemical and thermal stability in vivo, and biocompatibility [124, 143–147, 149, 150, 153, 155–157].

Not all titanium dioxide (TiO_2_) NPs are the same; they exist in distinct polymorphic forms, anatase, rutile, and brookite, each with unique physical and chemical properties. For instance, anatase exhibits a higher photocatalytic activity due to its optimal bandgap of 3.2 eV, while rutile, with a bandgap of 3.0 eV, is more stable under UV light. These differences make anatase more suitable for photocatalysis and drug delivery applications, while rutile is preferred for stability in coatings and sunscreens. The variations in crystal structure significantly impact the performance and potential applications of TiO_2_ NPs [109].

**Figure 7:**
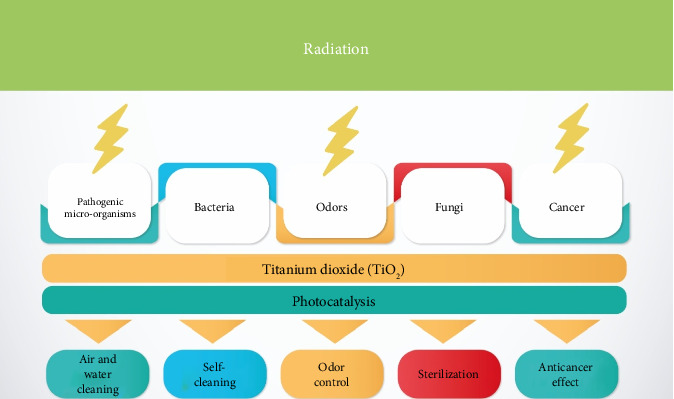
Representation of the effect of photocatalytic activation of TiO_2_ on the biological system (adapted from [111, 151]).

Titanium oxide, primarily found in its TiO_2_ form, is prevalent and has diverse applications, including food items, medicinal products, pharmaceuticals, and plastics [111, 148]. In biomedicine, NPs of titanium oxide (TiO_2_ NPs) are extensively utilized. These NPs generate ROS when photoexcited, which are known to damage or deactivate cancer cells.  Figure 7 shows the photogenerated process of TiO_2_, which is carried when the water and oxygen molecules of cells react with electrons in the conduction band and holes (h+) in the valence band, producing anionic oxygen and hydroxyl radicals that destroy biological systems like cancer cells [111, 151]. Likewise, TiO_2_ NPs recently have been described as inert in humans [113, 152]. Nevertheless, other studies have shown that TiO_2_ NPs are toxic via injection, inhalation, and ingestion and are characterized by inflammation and cardiac alteration function [115, 117, 154, 155]. Therefore, the treatment against dangerous cells seems to depend on variables such as cell type, size, concentration nanofiller, and surface properties.

### 4.5. Cerium Oxide Nanoparticles (CeONPs)

CeONPs exhibit remarkable antioxidant properties due to their ability to cycle between Ce^3+^ and Ce^4+^ oxidation states, allowing them to continuously scavenge ROS. This redox cycling mimics the activity of natural antioxidant enzymes, such as superoxide dismutase and catalase, enabling CeONPs to alleviate oxidative stress in various pathological conditions. This property makes CeONPs ideal for reducing damage to neuronal cells caused by ROS in the CNS. For example, in a study by [118], CeONPs demonstrated efficacy in mitigating inflammation and oxidative damage by scavenging ROS and inhibiting macrophage activation [118]. Additionally, their ability to be excreted rapidly minimizes long-term toxicity, making them promising candidates for clinical applications.

## 5. Discussion

Given the complexity and specialization of the CNS, disorders impacting it are equally complex, resulting in significant damage and behavioral disruptions. The existence of the BBB renders current treatment approaches for these disorders insufficient. Consequently, innovative strategies, such as those involving NPs, are essential for effectively treating neurological disorders. The use of metallic NPs for diagnosis or to enhance drug delivery across the BBB has garnered considerable interest because of their various advantages. However, they also have drawbacks that limit their practical application in medicine [104–106, 158–160].

Throughout this review, the potential benefits of metallic NPs have been consistently underscored. These advantages generally include precise targeting, noninvasiveness, biodegradability, stability, the capacity to penetrate the BBB, and control over drug loading and release [106, 160]. Currently, the treatment options for neurological disorders include surgery, radiotherapy, chemotherapy, and immunotherapy. In recent years, other approaches, such as gene therapy, hyperthermia therapy, and stem cell therapy, have also been utilized for treating these conditions [53, 65]. However, to achieve effective therapy, early diagnosis is crucial. The commonly used diagnostic techniques are imaging-based: CT, MRI, PET, and SPECT, which can sometimes support clinical decisions [107, 132, 133, 161–163]. Despite significant advancements in these techniques, the successful treatment of neurological disorders is still limited by several challenges associated with diagnostic imaging. These include (i) time-consuming processes that lack sensitivity, (ii) inadequate penetration and increased scattering in brain tissues during fluorescence imaging, (iii) the inability to monitor disease progression alongside drug treatment, (iv) toxic effects on adjacent healthy brain tissue due to the nonspecific targeting of radioactive tracers used in PET and SPECT imaging [134, 135, 164, 165].

Similarly, standard treatments also face numerous challenges such as: (i) their inability to cross the BBB, (ii) they result in inadequate drug uptake in neural tissue, (iii) they have suboptimal biocompatibility, (iv) they do not allow for targeted delivery and distribution, (v) they have poor solubility, (vi) they result in short half-lives and retention time, and (vii) they cause harmful effects on rapidly dividing healthy cells [53, 65].

To tackle these issues and ensure effective therapy, developing advanced methods that offer precise resolution, deep penetration, enhanced sensitivity, and the ability to monitor disease progression in real time while maintaining biosafety is crucial. In this context, ultrasmall multifunctional metallic NPs could play a significant role in biomedical imaging and the treatment of neurological diseases [1, 32, 33, 41, 42, 51, 63, 136, 137, 166, 167].

Despite the potential and promising results shown by studies, the field of NPs is still developing, with no system currently meeting clinical standards for successful translation in healthcare [53, 65]. The physicochemical properties that enable metallic NPs to be effective in neurological applications also contribute to their toxicity, presenting significant health risks. Additionally, the harmful effects of NPs can be markedly distinct compared to their larger, bulk forms. It is essential to fully understand these health concerns for NPs to be effectively utilized [138–140, 168–170]. Once they cross the BBB, metallic NPs accumulate in specific brain regions, impacting various brain cells, including neurons, astrocytes, and microglia. This can lead to changes in the structure or functionality of the neural system or induce further effects via glial activation and neuron–glial interactions. Adverse neurological effects may occur through several pathways such as oxidative stress, which causes cellular apoptosis and autophagy, immune reactions, and neuroinflammation, ultimately affecting BBB integrity [106, 141, 160, 171].

The neurotoxic effects of NPs primarily stem from the excessive production of ROS, which leads to oxidative stress. This situation then causes the release of cytokines, resulting in neuroinflammation and ultimately leading to neuronal death through apoptotic pathways. Certain metallic NPs, such as iron oxide, silver, gold, and titanium, are recognized for their potential neurotoxicity [141, 142, 171, 172]. Given the significant capabilities of metallic NPs as diagnostic and therapeutic tools for neurological disorders, it is vital to improve their functionalization and characterization continuously. Additionally, it is essential to thoroughly test and refine NP interactions in experimental settings to achieve optimal biocompatibility with cellular structures, thus reducing damage to healthy tissue and minimizing neurotoxic effects [1].

The neurotoxic effects of NPs are influenced significantly by their size, surface charge, and chemical coating. For instance, smaller NPs demonstrate higher cellular uptake but are more prone to induce oxidative stress and mitochondrial dysfunction, as observed with titanium dioxide NPs in neuronal PC-12 cells. PVP coatings have been shown to mitigate these effects by reducing oxidative stress and inflammation [173]. Similarly, the surface charge plays a critical role; positively charged NPs tend to interact more aggressively with cell membranes, leading to increased cytotoxicity compared to neutral or negatively charged variants [174]. These findings underscore the importance of NP engineering in minimizing neurotoxic effects for safe biomedical applications.

Substantial scientific advancements are required to surmount these complex challenges. Through dedicated research and development, nanotechnology can transition from laboratory settings to clinical applications, enabling effective and personalized diagnostic and therapeutic strategies.

## 6. Limitations and Future Perspectives

While nanotechnology has shown tremendous promise in biomedical applications, significant gaps remain in its clinical translation. A critical limitation is the scarcity of rigorous in vivo studies that evaluate NPs' long-term efficacy and safety. For example, many preclinical studies lack extended timelines to assess potential chronic toxicity, biodistribution, and systemic clearance, which are essential for regulatory approval and patient safety. Furthermore, large-scale, well-controlled studies replicating real-world clinical conditions are necessary to establish reproducibility and address variability observed in preclinical research. As highlighted by [175], CeONPs demonstrate potential for addressing oxidative stress but face challenges in biodistribution and toxicity, limiting their clinical adoption [175].

The future of nanotechnology in medicine lies in developing multifunctional NPs capable of precise targeting, sustained therapeutic release, and minimal off-target effects. Advances in NP engineering, such as hybrid nanoplatforms and responsive drug delivery systems, hold the potential to overcome current limitations. Furthermore, integrating artificial intelligence and machine learning into nanomedicine research can enhance design optimization and prediction of biological interactions. These advancements, coupled with robust long-term studies and standardized protocols, will be critical in ensuring nanotechnology's safe and effective translation from the lab to the clinic.

## 7. Conclusions

This review highlights recent advancements using NPs for diagnosing and treating neurological disorders. Metallic NPs are increasingly recognized as valuable tools for early disease detection due to their enhanced selectivity and sensitivity compared to conventional methods [124, 176]. The inherent characteristics of metals—like charge, stability, and high surface-to-volume ratio—enable nanoscale modifications that have pioneered new techniques for managing complex conditions such as cancer and neurodegenerative diseases [125, 177]. The capacity of metallic NPs to penetrate the BBB, which typically restricts drug delivery, has opened new pathways for accessing brain tissues, thereby improving both therapeutic interventions and diagnostic accuracy for neurodegenerative disorders.

Additionally, research indicates that metallic NPs may help prevent neuronal loss associated with neurodegenerative diseases such as AD [126, 178]. Conversely, research conducted in both in vivo and in vitro environments indicates that metallic NPs can disrupt neuronal communication and alter cellular homeostasis, potentially contributing to CNS disorders and exacerbating symptoms of AD [127, 179]. The impact of metallic NPs within the body varies based on their composition, concentration, and interaction with the patient's genetic makeup, influencing various outcomes including diagnosis, treatment efficacy, drug delivery, neurodegeneration, and neuroprotection [128, 180].

Many of these metallic NPs exhibit biocompatibility and can be adapted with various ligands and molecules to enhance their specificity and therapeutic efficacy. The optical, magnetic, and antioxidant characteristics of metallic NPs allow diverse therapeutic and diagnostic applications. Some of these particles can cross the BBB, a critical aspect in treating diseases that affect the CNS.

Despite these promising findings, most research involving metallic NPs against neurodegenerative diseases has been conducted in vitro or with animal models. These studies confirm the capability of metallic NPs to traverse the BBB effectively and delineate both their beneficial and adverse effects on the CNS.

Despite significant progress, the clinical application of metallic NPs remains constrained by challenges such as large-scale manufacturing, biocompatibility, and regulatory hurdles. A clear path forward involves addressing these issues through robust preclinical and clinical studies that assess long-term safety and efficacy. Furthermore, developing standardized protocols for NP synthesis and functionalization, alongside innovative strategies to mitigate potential toxicity, will be essential. The future of nanomedicine lies in integrating multifunctional NPs with enhanced precision, offering simultaneous diagnostic and therapeutic capabilities. Such advancements and interdisciplinary research efforts could revolutionize neurological treatments and bridge the gap between laboratory research and clinical implementation.

## Figures and Tables

**Table 1:** Application of metal nanoparticles (MNPs) in the treatment of neurological disorders.

Metal	Nanomaterial characteristic	Technic description	Aim/neurological disorder	Results	Reference
IONPs	DDNP-SPIONs, a carboxyl derivative of 1,1-dicyano-2-[6-(dimethylamino) naphthalene-2-yl] propene (DDNP) for functionalizing SPIO surfaces, 60–150 nm in diameter, linked with β-amyloid aggregates	Magnetic resonance microimaging in AD transgenic mice	Ultrasensitive nanoprobes for AD imaging	DDNP-SPIO nanoparticles may be useful for imaging Aβ plaques, aiding early Alzheimer's disease diagnosis and tracking drug therapy effects.	[82–84]

IONPs	USPIONs 10–140 nm with Fe-core chemically coupled with Aβ_1–42_	Magnetic resonance microimaging in AD transgenic mice	Identifying amyloid plaques in vivo through the use of magnetic resonance microimaging for AD diagnosis.	Following the injection of USPIO-Aβ1-42, it was possible to discern variances between AD transgenic mice and their wild-type counterparts. The potential of employing minimally invasive intravenous femoral injections to detect amyloid plaques in AD transgenic mice makes this approach suitable for long-term studies investigating AD development.	[82–85]

IONPs	Alginate-conjugated Fe_3_O_4_ NPs	Method of transportation: G23 peptide/receptor-mediated endocytosis. Anticancer drug (doxorubicin Dox) nanocarrier G23-alg-Fe_3_O_4_ NPs Tested on mice.	Brain tumor	Secure nanovehicle for brain tumor therapy. MRI contrast medium appropriate for both in vitro and in vivo diagnosis. Notable shrinkage in tumor dimensions noticed in treated mice over a span of 7 days.	[53, 65, 67, 82]

IONPs	Congo red/rutin MNPs 13 nm size	Method of transportation: penetration. In vivo, IV: APPswe/PS1dE9 transgenic mice brains.	AD	Congo red-labeled MNPs have been produced. Aβ aggregates can be identified using MRI. Drug release is controlled and reacts to H_2_O_2_, helping prevent oxidative stress in AD therapy.	[53, 65, 103, 108]

AuNPs	Transferrin-coated gold nanoparticles containing phthalocyanine 4 (Tf-coated AuNP with Pc 4).	In vitro study using human glioma U87 cells and colocalization immunofluorescence to visualize the Pc4 location. And photodynamic therapy (PDT).	Brain tumor	Using Tf_pep_ as the targeting agent enhances the specificity of AuNPs for cells that overexpress the transferrin receptor. Tf_pep_-AuNPs-Pc 4 effectively accumulate in orthotopic brain tumors with negligible accumulation in other areas.	[92, 105]

Au nanosphere	Coated with PEG and fluorescent tag (Pc4) 41 nm size BBB-crossing enhancer: Tf	Culture: U87 and U227 glioblastoma multiforme (GBM) cells in vitro. IV: U87 GBM orthotopic xenograft in mice in vivo. Imaging modality: Fluorescence microscopy.	Brain tumor	Tf conjugation increased AuNP uptake in GBM orthotopic xenografts compared to nonconjugated NPs. The Au nanospheres showed high specificity for brain tumors with minimal presence in other organs, regardless of Tf conjugation.	[71, 92, 94, 105]

Au nanosphere	Coated with PEG and fluorescent tag (Pc4) 41 nm size BBB-crossing enhancer: Tf, EGF (epidermal growth factor)	Culture: U87 and U227 glioblastoma multiforme (GBM) cell lines cultured in vitro. IV: In vivo administration in mice with U87 GBM orthotopic grafts. Imaging modality: Fluorescence microscopy.	Brain tumor	Double-targeted AuNPs cross the blood–brain barrier more effectively than their untargeted counterparts, leading to quicker and higher accumulation levels. Furthermore, these double-targeted nanoparticles exhibit reduced accumulation in critical organs when compared to single-targeted AuNPs.	[71, 93, 94, 106]

Au nanosphere	Coated with PEG and Gd tag, and doxorubicin 21 nm size BBB-crossing enhancer: TAT (transactivator of transcription)	Culture: U87, U251, GBM 43, and GL261 glioblastoma multiforme (GBM) cell lines cultured in vitro. IV: In vivo studies involve U87 GBM orthotopic xenografts implanted in athymic nude mice. Imaging modality: MRI.	Brain tumor	TAT-AuNP-Gd conjugates result in a more pronounced and sustained enhancement of brain tumors, with signals remaining detectable 24 h postadministration. TAT-AuNPs conjugated with doxorubicin (Dox) successfully cross the BBB and are preferentially absorbed by tumor cells. The use of TAT-AuNPs conjugated with Dox markedly enhances survival in mice compared to treatment with Dox alone or TAT-AuNPs alone. Additionally, TAT-AuNPs, whether conjugated with Dox or gadolinium (Gd), do not produce adverse effects in vivo.	[10, 13, 71, 94]

AuNPs	Positively charged poly(allylamine hydrochloride)-coated AuNPs.	In vitro research utilizing α-synuclein, an amyloid protein prone to aggregation, which has been proposed as a potential contributor to PD.	PD	α-Synuclein assumes a random orientation on PAH-coated AuNPs. A consistent change in the conformation of α-synuclein across all concentrations of PAH AuNPs indicates that the α-synuclein bound to the nanoparticles induces conformational changes in free α-synuclein.	[94, 107]

AuNPs	Chitosan-modified AuNPs, functionalized with cationic groups, are capable of binding plasmid DNA and are involved in nerve growth factor receptor–mediated interactions (CTS–AuNP–pDNA–NGF composites). These composites range in size from 5 nm to 10 nm.	In vitro: AuNP composite was transfected into cells via endocytosis to inhibiting the apoptosis of PC12 cells. Treated with different concentrations of 1-methyl-4-phenylpyridinium (MPP^+^). In vivo: IV treated C57bl/6 mice.	PD	In both in vitro and in vivo models, α-synuclein expression was reduced in PC12 cells treated with AuNP composites and MPP+ compared to those treated solely with MPP+. The AuNP composites were internalized into cells through endocytosis, leading to the suppression of α-synuclein expression, which in turn inhibited apoptosis in PC12 cells and dopaminergic neurons of the substantia nigra striatum.	[43, 55]

AgNPs	AgNPs are spherical and homogeneous in size, with a diameter of approximately 10 nm.	Solutions containing two different silver compounds were administered to two separate groups of Winstar rats via a gastric tube. The prescribed amount was established at 0.2 mg/kg of body weight daily, administered over a span of 14 days. One group received silver citrate, while the other was given regular AgNPs.	Investigate the biochemical parameters related to oxidative stress in myelin obtained from the brains of adult rats that have been exposed to a minimal dosage of AgNPs.	Following ingestion, AgNPs are found to gather in the brains of the subjected rats. Oxidative stress is pinpointed as a crucial factor in the neurotoxicity triggered by AgNPs, especially emphasizing the effect of a minimal dose on the protein and lipid constituents of myelin membranes, which could potentially influence the structure of myelin sheaths. The breakdown of myelin could result in the dysmyelination or demyelination of axons, the deterioration of demyelinated nerve fibers, and the interruption of neuronal transmission	[109, 110]

AgNPs	The levels of NPs were established at 2 and 4 mg/L. AgNPs displayed a particle dimension of under 100 nm, a surface area measuring 5.0 m^2^/g, a density value of 10.49 g/cm^3^, and a purity percentage of 99.5%.	The investigation made use of distinct NP concentrations, specifically set at 2 and 4 mg/L.	The research investigated the immediate and prolonged toxicity of AgNPs on the brain tissue of two types of fish, *Oreochromis niloticus* and *Tilapia zillii*.	The concentration of Ag in the brain tissue of *O. niloticus* and *T. zillii* increased with exposure to AgNPs. This increase was particularly marked in fish exposed to a higher concentration of 4 mg/L compared to those exposed to 2 mg/L and the unexposed control group. Regarding glutathione (GSH) and total glutathione (tGSH) in the brain tissues of these fish, no observable change occurred in the levels for the group exposed to 2 mg/L of AgNPs. However, a significant reduction in GSH and tGSH levels was evident in the group exposed to 4 mg/L, when compared to both the control group and the group exposed to the lower concentration. As for malondialdehyde (MDA) levels remained stable in the brains of fish exposed to the lower concentration of AgNPs. In contrast, there was a marked increase in MDA levels in the brains of fish exposed to the highest concentration of AgNPs. This rise is likely due to the depletion of the antioxidant defense mechanism, as identified in this study. Additionally, the formation of ionic Ag from AgNPs in the brain tissue contributes to the disruption of cellular and mitochondrial membranes, predominantly through the oxidation of polyunsaturated fatty acids, resulting in increased MDA production. Among the various mechanisms proposed to explain the toxicity of AgNPs, the stimulation of reactive oxygen species (ROS) synthesis stands out as particularly significant.	[111, 112]

Synthesized AgNPs (2 different samples)	Nanoscale silver particles (nano-Ag) were traditionally produced by reacting silver nitrate (AgNO_3_) with sodium borohydride (NaBH4). Subsequently, some of these particles were encapsulated using green tea extracts, while another was encapsulated with glutathione.	This study investigates the initial effect on barrier transport by assessing whether nano-Ag particles, synthesized via “green” methods and coated with green tea polyphenols (GT) or glutathione (GSH), can alter the permeability of human intestinal epithelial (Caco-2) or rat brain endothelial (RBEC4) barrier cells.	Effect on rat brain endothelial (RBEC4) barrier cells.	Research has shown that traditionally produced nano-Ag particles without a coating can cause stress in the BBB. This stress leads to the release of inflammatory substances in rat brain endothelial cells (RBEC4), which in turn increases their permeability. Measurements of transepithelial resistance (TER) have indicated that these materials significantly alter the permeability of cell membranes within just 15 min of exposure, particularly affecting the cell membrane of RBEC4, comprised of vascular endothelial cells from rat brains. Additionally, studies suggest that both traditional and environmentally friendly coated nanosilver particles influence membrane permeability in intestinal epithelial cells and BBB endothelial cells, and are linked to oxidative stress-driven neurotoxic effects.	[113, 114]

AgNPs	AgNPs ranging from 3 to 5 nm were produced using a physical method, devoid of any surfactants or stabilizers.	This study investigated how AgNPs might affect genes associated with inflammation and neurodegenerative diseases. It focused on murine brain astrocytes, microglial BV-2 cells, and neuronal N2a cells. Researchers introduced 3–5 nm AgNPs into the culture medium and exposed these cell types to the nanoparticles in vitro for 24 h.	Assess the potential impacts on gene expression related to inflammation and neurodegenerative diseases.	The processing of glutathione plays a crucial role in shielding cells from oxidative damage. Following the administration of 25-nm AgNPs, there is a notable alteration in the gene expressions linked to oxidative stress in the caudate, frontal cortex, and hippocampus of male C57BL/6N mice. AgNPs can penetrate mouse neural cells, inducing the release of proinflammatory cytokines and enhancing the accumulation of Aβ amyloid due to shifts in gene expression associated with inflammation, oxidative stress, and Aβ degradation. These results indicate that the neuroinflammatory reactions and Aβ accumulation caused by AgNPs could contribute to the progression of neurodegenerative diseases.	[115, 116]

Polyvinylpyrrolidone (PVP)–coated AgNPs	AgNPs coated with PVP were produced using glucose to reduce Ag + ions, with PVP present during the reaction.	This study analyzed the effects of exposing brain cells to AgNPs, with a specific focus on the impact of AgNPs on astrocytes.	Investigate the implications for astrocytes, as they are the initial cells that a toxic particle encounters after crossing the BBB.	Cultured astrocytes rapidly accumulate AgNPs through a process that is dependent on time, concentration, and temperature, likely involving endocytotic mechanisms. Despite the swift increase in their silver content, astrocytes show no adverse effects after exposure to AgNPs, suggesting they do not release significant amounts of harmful Ag+ ions from the accumulated nanoparticles. The ability to effectively accumulate silver and their robust resistance to the toxicity of AgNPs suggest that astrocytes are particularly well-equipped among various brain cell types to serve as a reservoir for potentially toxic metals.	[108, 117–121]

Modified AgNPs (AgEO and Ag citrate)	AgEO-NPs have a size range of 2–85 nm, while Ag citrate-NPs vary between 5 and 65 nm.	The experiment utilized primary porcine brain capillary endothelial cells and primary capillary choroid plexus epithelial cells in an in vitro setting. Researchers assessed the harmful effects of nanoparticles by examining changes in the integrity of cell membranes, cellular shape, functionality of cellular barriers, levels of oxidative stress, and inflammatory activity.	The research investigates the impact of AgEO and Ag citrate-NPs on the brain's protective barriers.	The observed toxicity of AgNPs is closely linked to the presence of reactive oxygen and nitrogen species (RONS). As the concentration of AgNPs increases, there is a disruption of barrier function, followed by DNA damage and, at the very least, a reduction in cell viability. Notably, the surface coating of AgNPs plays a critical role in determining their toxic potential. The impact of surface charge and coating on AgNPs was investigated. Specifically, two types of AgNPs were compared: AgEO-NPs (coated with ethylene oxide) and Ag citrate-NPs (coated with citrate). The Ag citrate-NPs, which exhibit a negative charge, had a less pronounced effect on the BBB and the blood–cerebrospinal fluid barrier in vitro. This variance was observable in several aspects, such as barrier dysfunction, production of reactive oxygen and nitrogen species (RONS), DNA damage, and the control of inflammation markers. Remarkably, although the primary brain microvascular endothelial cells (PBCEC) absorbed AgEO- and Ag citrate-NPs at similar rates, their responses to these nanoparticles varied. It is hypothesized that the reduced toxicity of Ag citrate-NPs may be attributed to a lower release of Ag+ ions from their surface. Cytotoxic effects of silver nanoparticles lead to increased barrier permeability and disrupted cell borders, as evidenced by staining for occludin. AgEO-NPs caused significant damage to the tight junction strands, whereas Ag citrate-NPs did not affect DNA integrity. While this study does not directly relate to brain health, it provides valuable insights into the toxic effects of AgNPs. Understanding these mechanisms is crucial for assessing the safety of nanomaterials and their potential impact on biological barriers.	[110, 122]

AgNPs	AgNPs and silver microparticles were employed.	In the study, AgNPs were subcutaneously injected into rats. Subsequently, the brains of the rats were obtained at predetermined time points for ultrastructural observation and detection of silver levels.	AgNPs were investigated for their accumulation in the brain, as well as their effects on the BBB.	Swelling of astrocytes outside the BBB was observed in rats exposed to AgNPs. Additionally, nanometer-sized, electron-dense, spherical particles were identified within the vascular endothelial cells of the compromised BBB, suggesting the presence of silver nanoparticles. This indicates that silver nanoparticles can exert neurotoxic effects, leading to neuronal degradation and thrombolysis of the cell membrane. Consequently, silver nanoparticles may transfer from damaged neurons to affect neighboring cells. Given that silver nanoparticles can persist in the brain for extended periods, their accumulation could lead to significant pathological alterations in the brain, ultimately increasing the incidence of neuronal necrosis.	[112, 123]

Citrate-capped AgNPs	AgNPs were produced using the chemical bath reduction technique, employing trisodium citrate (Na_3_C_6_H_5_O_7_) as the reducing agent.	The in vitro uptake and intracellular transformation of citrate-capped AgNPs by microglia and their impact on microglial inflammation and related neurotoxicity were analyzed.	The investigation focuses on how AgNPs affect microglial inflammation, aiming to thoroughly assess their neurotoxic potential and the associated risks of neurodegenerative diseases.	Silver ions (Ag+) engage with cellular membranes, resulting in diminished integrity and enhanced permeability of the membranes. These ions also interact with thiol groups in proteins, leading to impaired protein function. Furthermore, they form bonds with DNA, causing damage that triggers cell apoptosis.	[114, 124]

AgNPs	AgNPs exhibit a size range between 50 and 100 nm, while silver microparticles (SMPs) are sized from 2 to 20 μm.	A model of the BBB in vitro has been created using co-cultures that combine rat brain microvessel vascular endothelial cells with astrocytes.	This study aimed to explore how AgNPs are distributed after crossing the BBB.	In the group exposed to AgNPs, endothelial cells within the BBB showed signs of degeneration, including extensive vacuolation and some necrosis of cellular organelles. Observations also included degranulation, partial disintegration of the rough endoplasmic reticulum, and mitochondrial enlargement. AgNPs accounted for 34.12% of the total introduced silver mass when interacting with BBB cells. It is reasonable to propose that AgNPs interact with certain cellular structures like mitochondria and the rough endoplasmic reticulum in endothelial cells. Following this interaction, AgNPs accumulate in the cytoplasm, leading to ultrastructural and functional alterations in these organelles. These changes induce apoptosis of the endothelial cells, disintegration or dissolution of the cell membrane, and weakening of the tight junctions between endothelial cells. Consequently, these structural and functional disruptions enable free AgNPs to traverse the BBB through the resulting gaps.	[116, 125]

Polyvinylpyrrolidone (PVP)–coated AgNPs.	AgNPs, stabilized with PVP, have a nominal diameter of 50 nm.	The investigation was conducted using an in vitro model of the blood–brain barrier (BBB) to examine its impacts. The research spanned two intervals, specifically 24 and 48 h.	Examine the effects of AgNPs' toxicity on the BBB.	The transcytosis of AgNPs from endothelial cells to astrocytes has been documented. Transmission electron microscopy data revealed significant accumulation of AgNPs within endothelial cells at both the 24- and 48-h marks. In contrast, astrocytes exhibited fewer vacuoles containing AgNPs compared to their endothelial counterparts. It was noted that only a limited number of AgNPs successfully penetrated the astrocytes from the endothelial cells. This study highlights a potentially novel cellular mechanism, suggesting that the BBB may return to a normal state from an inflamed condition after initial damage. This finding is pivotal as it illuminates the dynamic cellular interactions of the BBB in response to AgNPs over time. Initially, within the first 24 h, certain proteins were either downregulated or upregulated. By the 48-h mark, most of the essential proteins involved in BBB protection had returned to normal levels.	[119, 126]

Anatase TiO_2_	Doses of 2.5, 5, and 10 mg/kg of anatase TiO_2_ were administered to mice.	The mice were subjected to exposure to anatase TiO_2_. Each group received varying dosages of 2.5, 5, and 10 mg/kg. The exposure period to the TiO_2_ lasted for 90 days.	The study evaluated the effects of anatase TiO_2_ on mouse brains.	After a 90-day period, the analysis revealed that mice exhibited neurogenic disease states. This finding suggests that exposure to the anatase form of TiO_2_ could potentially lead to neurodegenerative diseases in animals. Furthermore, there exists a possibility that humans may develop similar conditions following exposure to this form of TiO_2_.	[120, 127]

Anastase TiO_2_ (Degussa anatase TiO_2_)	The dose of nanoparticles used for this experiment was about 2.5–120 ppm of anatase TiO_2_ with an exposure time of 16 and 18 h	A nanomaterial known as Degussa P25, which has been thoroughly characterized in terms of its physical and chemical properties, was introduced to brain microglia (BV2).	This research aimed to ascertain the impact of TiO_2_ on brain microglia (BV2).	The biological response of BV2 microglia to nontoxic (2.5−120 ppm) concentrations of P25 was marked by a rapid (< 5 min) and sustained (120 min) release of reactive oxygen species. This release pattern suggests that P25 initiated an immediate “oxidative burst” in the microglia and interfered with mitochondrial energy production.	[121, 128]

Anatase and rutile TiO_2_	The dose of nanoparticles used for this experiment was 20 mg/cm^2^ with an exposure time of 2, 4, 6, and 24 h	The study aimed to evaluate the toxicological impact of TiO_2_ nanoparticles on glial cells, specifically rat-derived C6 cells and human-derived U373 cells.	This research aimed to explore the potential of TiO_2_ to trigger oxidative stress and influence other areas of the brain.	TiO_2_ nanoparticles elicited significant oxidative stress in both types of glial cells, driving alterations in the lipid peroxidation and cellular redox state. This was linked with an upsurge in the expression of enzymes such as catalase, glutathione peroxidase, and superoxide dismutase 2. The nanoparticles also induced morphological transformations, mitochondrial damage, and an elevation in mitochondrial membrane potential, all indicative of toxicity. The cytotoxic influence of TiO_2_ nanoparticles on glial cells was evident. Further research is warranted given the potential of TiO_2_ to inflict brain damage and pose health risks.	[122, 129]

Anatase TiO_2_	The dose of nanoparticles was 10 g/mg with an exposure of 1 h	Mitochondria were extracted from the lung tissue of rats and subjected to exposure of 10 μg of TiO_2_ nanoparticles (with a particle size of less than 25 nm) per mg of protein for a duration of 1 h.	The study aimed to test the hypothesis that the effects of TiO_2_ nanoparticles are tied to mitochondrial dysfunction and an imbalance in redox reactions.	The findings indicated that TiO_2_ nanoparticles lead to a decrease in NADH levels and cause impairment in mitochondrial membrane potential (ΔΨm) and overall mitochondrial function. This is accompanied by the generation of reactive oxygen species (ROS) during the process of mitochondrial respiration.	[123, 130]

CeO_2_	The dose of nanoparticles was 0.1 mg/kg CeO_2_ in 0.5 mL phosphate-buffered saline	10 rats were stereotaxically injected with 10 μg of 6-hydroxydopamine hydrobromide (6-OHDAHBr) dissolved in 2 μL of vehicle into the left striatum of the brain. Subsequently, the rats were given an injection into the peritoneal membrane of low doses of CeO_2_ nanoparticles.	The main objective is to determine the neurological protective effect of CeO_2_ metal nanoparticles in diseases such as PD, multiple sclerosis, and AD.	CeO_2_ nanoparticles can neutralize almost all varieties of reactive species, have remarkable regenerative capacity, and can cross the BBB due to their minute size.	[131]

## Data Availability

This is a review article and does not include any original data. All data referenced in this manuscript are publicly available from the sources cited in the reference list.
