# Correction to “Metallic Nanoparticles Applications in Neurological Disorders: A Review”

**DOI:** 10.1155/ijbm/9871908

**Published:** 2026-03-05

E. Ibarra‐Ramírez, M. Montes, R. A. Urrutia, et al., “Metallic Nanoparticles Applications in Neurological Disorders: A Review,” *I. J. of Biomaterials*, 2025:4557622:30, https://doi.org/10.1155/ijbm/4557622.

In this article titled “Metallic Nanoparticles Applications in Neurological Disorders: A Review,” the affiliations for the author “Edwin A. Segura González” were omitted in error. The corrected author list and affiliation list are below:

Ernesto Ibarra‐Ramírez^1,2,3,4^, Melissa Montes^2^, Roger Alexei Urrutia^3^, Diego Reginensi^1,2,5,6^, Edwin A. Segura González^1,3,7^, Luis Estrada‐Petrocelli^2,6^, Alexandra Gutierrez‐Vega^8^, Abhishek Appaji^9^, and Jay Molino^1,6^

^1^Faculty of Biosciences and Public Health, Specialized University of the Americas (UDELAS), Panama City, Panama

^2^Faculty of Engineering, Latin University of Panama (ULATINA), Panama City, Panama

^3^Faculty of Engineering, Architecture and Design, Interamerican University of Panama (UIP), Panama City, Panama

^4^Faculty of Electrical Engineering, Technological University of Panama (UTP), Panama City, Panama

^5^School of Medicine, University of Panama (UP), Panama City, Panama

^6^National Secretariat of Science, Technology and Innovation (SENACYT)—National Research System (SNI), Panama City, Panama

^7^School of Industrial Technology, Specialized Higher Technical Institute (ITSE), Panama City, Panama

^8^Department of Biomedical Engineering, University of Arkansas, Arkansas, Fayetteville, USA

^9^Department of Medical Electronics Engineering, B.M.S. College of Engineering, Bengaluru, India

We apologize for this error.

